# Charon tectonics

**DOI:** 10.1016/j.icarus.2016.12.018

**Published:** 2016-12-16

**Authors:** Ross A. Beyer, Francis Nimmo, William B. McKinnon, Jeffrey M. Moore, Richard P. Binzel, Jack W. Conrad, Andy Cheng, K. Ennico, Tod R. Lauer, C.B. Olkin, Stuart Robbins, Paul Schenk, Kelsi Singer, John R. Spencer, S. Alan Stern, H.A. Weaver, L.A. Young, Amanda M. Zangari

**Affiliations:** a Sagan Center at the SETI Institute, 189 Berndardo Ave, Mountain View, California 94043, USA; b NASA Ames Research Center, Moffet Field, CA 94035-0001, USA; c University of California, Santa Cruz, CA 95064, USA; d Washington University in St. Louis, St Louis, MO 63130-4899, USA; e Massachusetts Institute of Technology, Cambridge, MA 02139, USA; f Johns Hopkins University Applied Physics Laboratory, Laurel, MD 20723, USA; g National Optical Astronomy Observatories, Tucson, AZ 85719, USA; h Southwest Research Institute, Boulder, CO 80302, USA; i Lunar and Planetary Institute, Houston, TX 77058, USA

**Keywords:** Pluto, Charon, Geological processes, Tectonics, Image processing

## Abstract

New Horizons images of Pluto’s companion Charon show a variety of terrains that display extensional tectonic features, with relief surprising for this relatively small world. These features suggest a global extensional areal strain of order 1% early in Charon’s history. Such extension is consistent with the presence of an ancient global ocean, now frozen.

## 1. Introduction

Charon, Pluto’s large companion, has a variety of terrains that exhibit tectonic features. We present observations of them here from New Horizons’ Long-Range Reconnaissance Imager (LORRI, Cheng et al., 2008) and the Multi-spectral Visible Imaging Camera (MVIC) on the Ralph instrument (Reuter et al., 2008). This work analyzes these observations and measures tectonic strike, estimates elastic thickness, compares these features to those seen on Pluto, and concludes that Charon has undergone global extension.

**Fig. 1:**
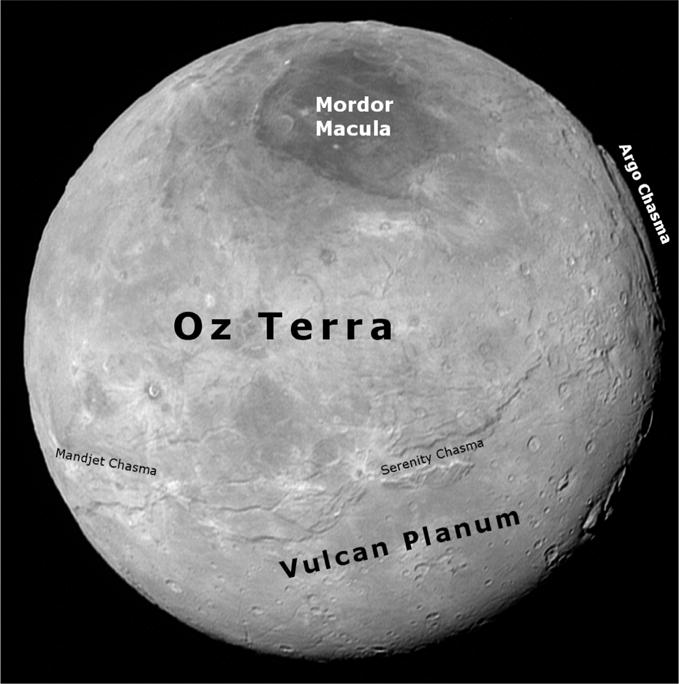
This is the last LORRI image that captures the whole disk of Charon and what is referred to as the New Horizons encounter hemisphere, roughly centered at 350° longitude, 40° latitude. Informal names of some features are provided, for more informal named features see Moore et al. (2016a). This is the C_LORRI_FULLFRAME_1 observation (LOR_029914776, ~2.4 km/pixel) which has been deconvolved to bring out features.

The Pluto-facing hemisphere that New Horizons (Stern et al., 2015) observed at high resolution has two broad provinces: the relatively smooth plains of Vulcan Planum^1^ in the southern part of the encounter hemisphere (Spencer et al., 2016; Moore et al., 2016a), and the zone north of Vulcan Planum, which we describe here, and informally named Oz Terra. This latter zone is characterized by grooves, graben, and scarps, which are signs of extensional tectonism (Fig. 1). The border between these two provinces strikes diagonally across the encounter hemisphere from as far south as −19° latitude in the west (~285° E longitude) to 26° in the east (~30° E longitude). We define longitude and latitude on Charon according to the right hand rule and follow the recommendations of Zangari (2015). Charon’s north pole points in the direction of the angular momentum vector and longitudes increase to the east. Charon’s prime meridian is the sub-Pluto longitude. Pluto’s pole is defined by Archinal et al. (2011a, 2011b).

**Fig. 2:**
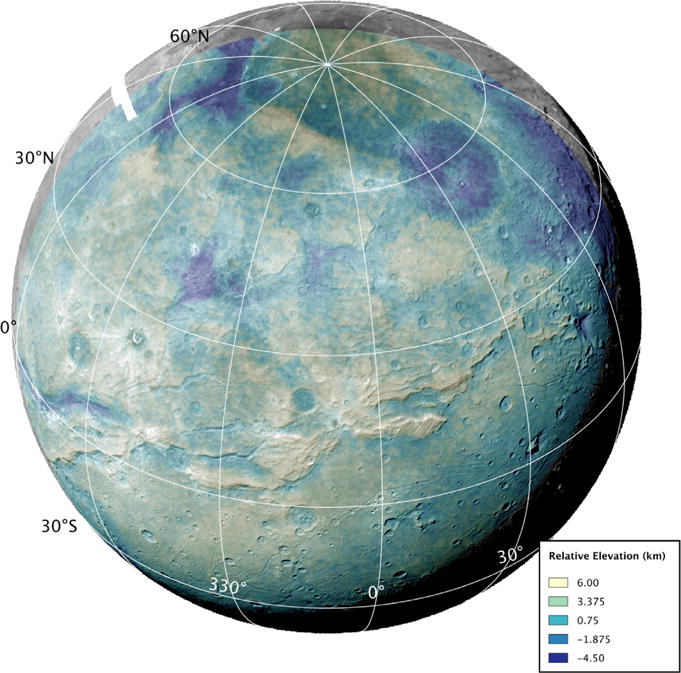
Charon terrain model in orthographic projection created from the PELR_C_LORRI and PEMV_C_COLOR_2 observations.

**Fig. 3:**
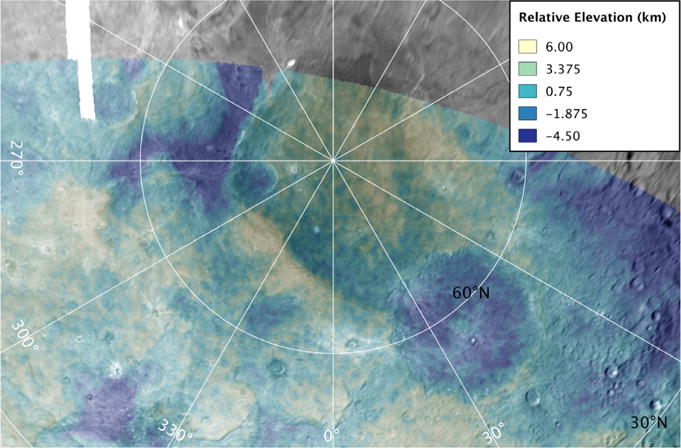
Polar stereographic view of Charon’s north pole created from the PELR_C_LORRI and PEMV_C_COLOR_2 observations.

North of the Vulcan Planum boundary with Oz Terra, the terrain is exceptionally rugged (Fig. 2) and contains a network of fault-bounded troughs and scarps in the equatorial to middle latitudes (Moore et al., 2016a). This transitions northward and over the pole to the visible limb into an irregular zone where the fault traces are neither so parallel nor so obvious, but which contains irregular depressions and other large relief variations (Fig. 3).

The lower-resolution views of the non-encounter hemisphere (Fig. 10) are also suggestive of other potential large ridges and troughs, indicating that the tectonic expressions we see so well on the encounter hemisphere likely extend around Charon.

## 2. Observations

There is a visually obvious belt of fractures just above the Vulcan Planum boundary that are highlighted by the solar illumination angle at these middle latitudes. The fault structures continue northwards from this zone, but are less obvious due to the decreasing illumination angle and changing style of tectonic deformation. We define three different zones in Oz Terra: low-latitude scarps and chasmata, mid-latitude scarps and crustal blocks, and high-latitude depressions and ridges. Although it is unknown if the tectonic features observed in Oz Terra are limited to the encounter hemisphere or if they extend around Charon and represent global latitudinal tectonic zones, there does seem to be some evidence for tectonic features on the non-encounter hemisphere (Fig. 10). There is no information on structures southward of −30°; due to the inclination of the Pluto system to its orbit about the Sun, all of that territory was in darkness during the New Horizons flyby.

New Horizons obtained extensive stereo coverage of the encounter hemisphere, and we used tested stereogrammetric methods after Schenk et al. (2004) to derive terrain from these images. The best global terrain data was generated from the PELR_C_LORRI and PEMV_C_COLOR_2 observations (Figs. 2 and 3) upon which our topographic interpretations are based. This terrain model has an approximate ground scale of 4.4 km/pixel, and an expected vertical precision of 1.2 km. We also used a terrain model created from the PEMV_C_COLOR_2 and PEMV_C_MVIC_LORRI_CA observations (Fig. 8). This terrain model also has an approximate resolution of 4.4 km/pixel but has an improved expected vertical precision of 0.3 km due to better parallax angle of the two images with each other.

Most of the highest-resolution images presented here were obtained with the New Horizons LORRI camera. The LORRI point spread function has a full width at half maximum (FWHM) of ~2 pixels, which meets its specifications (Cheng et al., 2008), but can cause noticeable blurring. This can be improved through (Lucy, 1974; Richardson, 1972) deconvolution, and the images for which this has been applied are noted.

### 2.1. Low-latitude scarps and chasmata

**Fig. 4:**
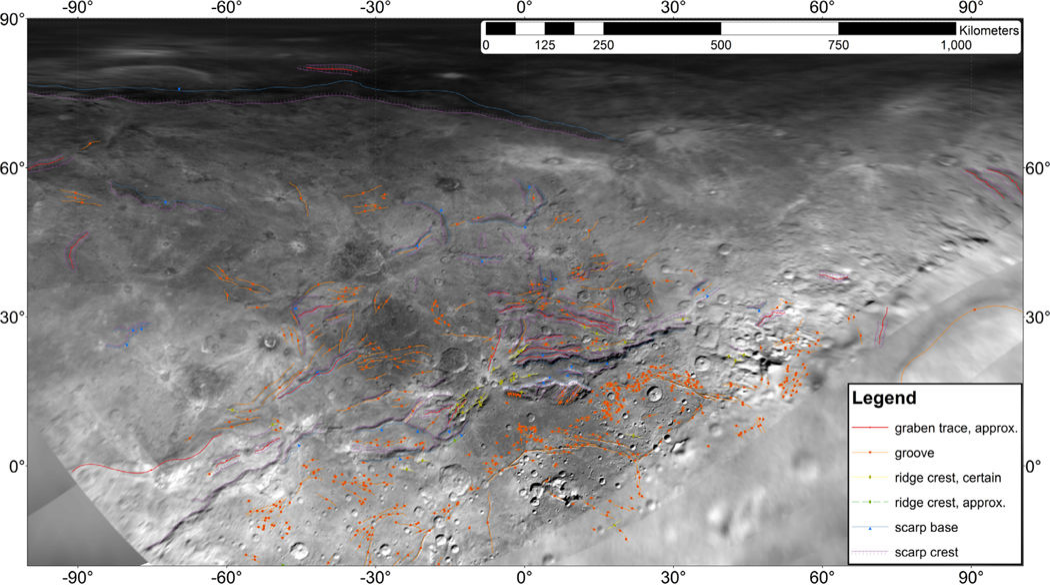
Charon tectonic features mapped in this work overlain on a mosaic of LORRI images.

**Fig. 5:**
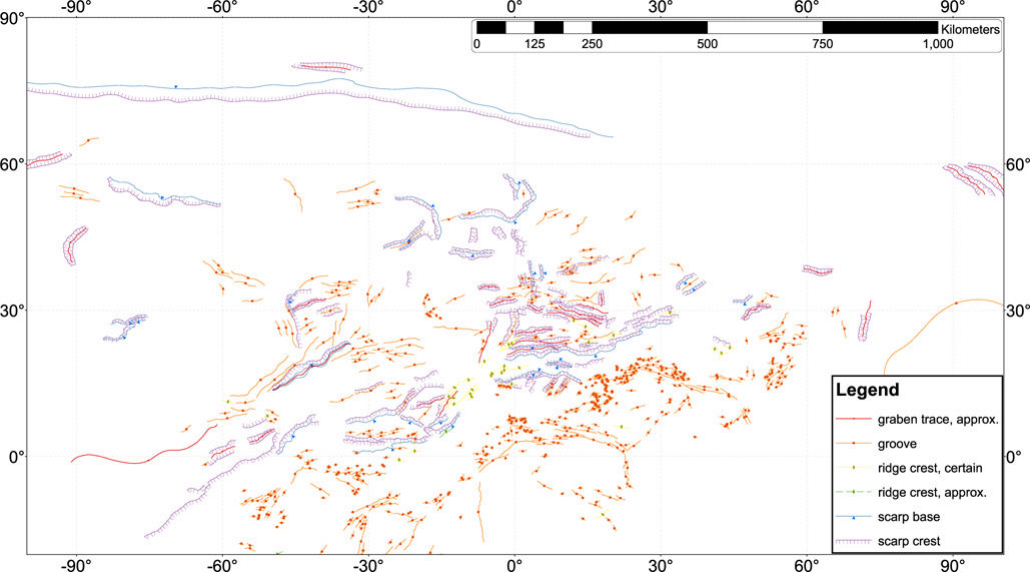
The same tectonic structures as mapped in Fig. 4 but without the underlying mosaic.

In the low-latitude zone of Oz Terra, there are a number of scarps and chasmata that generally parallel the strike of the northern margin of Vulcan Planum (Figs. 4 and 5).

The chasmata appear to be fault-bounded depressions, or graben, resulting from extension of the crust. In this region there are far more scarps that appear to be the trace of a single normal fault rather than a graben set, and these have a variety of expressions.

**Fig. 6:**
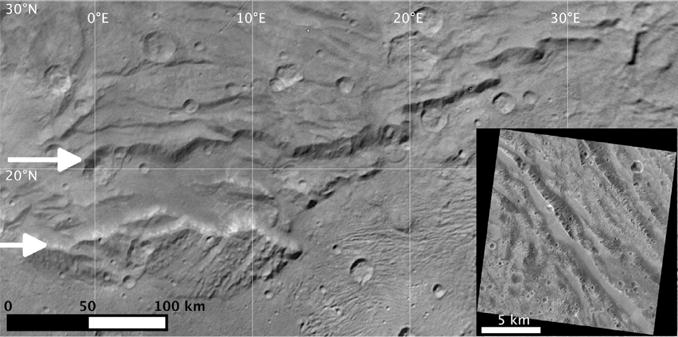
Serenity Chasma. White arrows point to the north and south rims of Serenity Chasma on this mosaic of several LORRI images (primary image is LOR_0299175682, deconvolved to bring out features). The surface beyond the northern rim of Serenity Chasma displays a series of small-relief mostly east-west faults parallel to the chasma rims, and the inset to the lower right is a Galileo image (GO_0023:[G28.GANYMEDE.C055244]3639R.IMG) of dark terrain on Ganymede’s Nicholson Regio, which may be showing similar faulting patterns; note scale difference.

There are small relief, parallel structures (Fig. 6) that are reminiscent of grooved terrain on other icy satellites (Pappalardo et al., 1998), which result from extensional instabilities and bookshelf faulting (Bland and McKinnon, 2015).

The largest scarps, with several kilometers of relief, face south and delineate the border with Vulcan Planum.

#### 2.1.1. Serenity Chasma

Serenity Chasma (Fig. 6) is 40–50 km wide rim-to-rim and is over 200 km long, with a maximum relief upwards of 5 km. At its eastern end where the southern rim terminates against the Vulcan Planum boundary, its northern rim continues another 200 km east as a south-facing scarp and becomes segmented by fault ramps.

#### 2.1.2. Mandjet Chasma

**Fig. 7:**
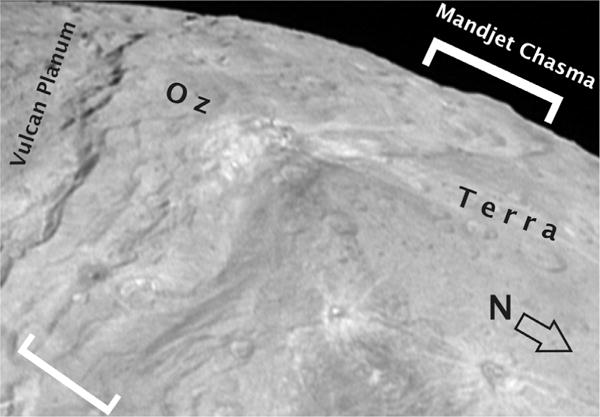
Perspective view of Mandjet Chasma as taken by LORRI (LOR_0299168968, deconvolved to bring out features) looking westward to the limb, North is to the lower right. Mandjet starts in the lower left of the frame and extends to the limb in the upper right (denoted by brackets). The scarp along the left side of the frame south of Mandjet bounds Vulcan Planum.

Mandjet Chasma (Fig. 7) is at least 450 km long and typically 30 km across. It was not identified in Stern et al. (2015) because it does not have a sharp topographic rim. It was identified in initial stereoscopic topography (Moore et al., 2016a), and its rim is a gentle slope as its walls transition from the surrounding plains and then plunge 5–7 km to its floor.

#### 2.1.3. Argo Chasma

Argo Chasma was clearly observed for the last time on Charon’s limb (Fig. 1), allowing us to determine that it was at least 5 km deep (Stern et al., 2015; Moore et al., 2016a). The strike of Argo Chasma may at first seem like it is at a steep angle with respect to the dominant pattern of strike across the encounter hemisphere, but that is because of a combination of the viewing geometry, its latitude, and its presentation along the limb in that observation. It can be seen in the maps of Figs. 4 and 5 on the eastern edge of the map. It is mapped as a long orange line just above the legend (around 90° E, 30° N), and it has a strike very similar to the strike of other structures.

### 2.2. Mid-latitude scarps and crustal blocks

**Fig. 8:**
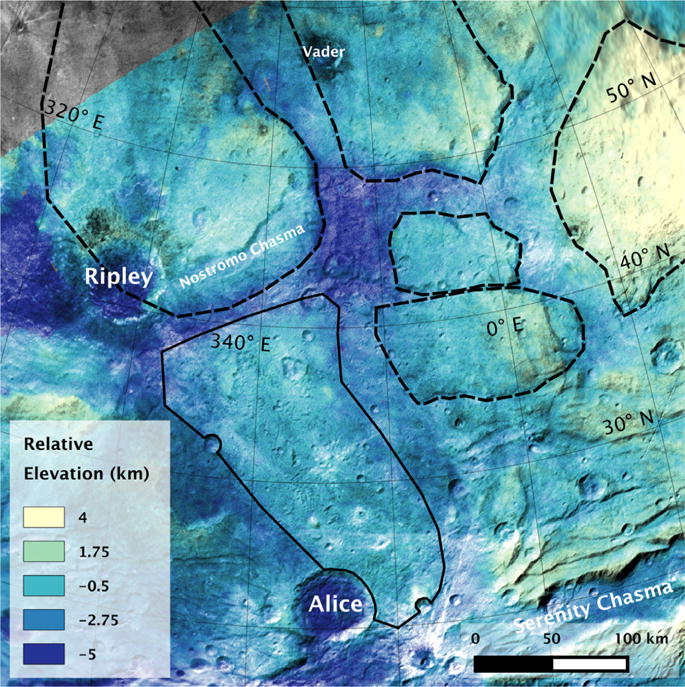
Topographic map of the mid-latitude scarps and crustal block region. What appear to be coherent crustal blocks bounded by scarps in this region are outlined with a dashed line. An elevated region that does not have the steeper sides of the crustal blocks in this region is outlined with a solid line. It may be a Vulcan Planum outlier. This is a mosaic of LORRI images from the C_LEISA_HIRES observation overlain by topography created from the PEMV_C_COLOR_2 and PEMV_C_MVIC_LORRI_CA observations.

North of the main belt of chasmata and scarps, the smaller ridges are not present, but there are several areas bounded by large continuous scarps that appear to be intact crustal blocks several hundred kilometers across. The ~400 m/pixel strip of C_LEISA_HIRES shows several well-defined blocks (Fig. 8) and there are indications of others in the images and digital terrain data (Fig. 2) in these latitudes.

These crustal blocks are characterized by having fewer small tectonic features (although that could be a combination of the phase angle and resolution decreasing northward) and instead can show a few very large bounding faults. The low boundaries between these competent crustal blocks have an elevation difference of a kilometer or two.

### 2.3. High-latitude depressions and ridges

**Fig. 9:**
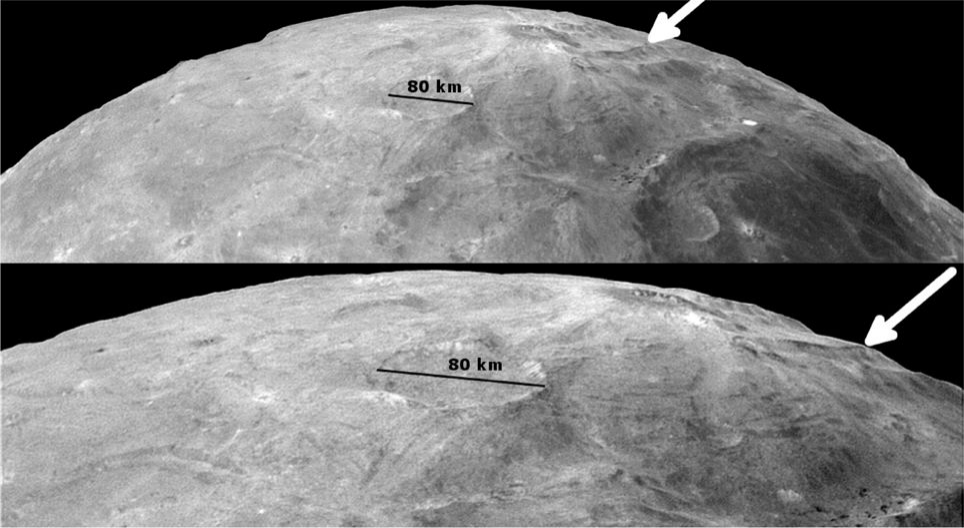
These images show the northern limb of Charon and its polar region to be irregular and displaying a large amount of relief. In the center and right, just below the limb in both images, scarps can be seen with dark features at their crests (the same scarp is identified with an arrow in each image). These are not shadows as these scarps face the Sun. The upper image is a mosaic of LORRI images from the C_LEISA_LORRI sequence. The dark Mordor Macula can be seen in the lower right. The lower image is a deconvolved version of LOR_0299175565 taken after the C_LEISA_LORRI sequence when the spacecraft was closer to Charon. The diameter of Pirx crater is called out in both images.

The north polar region outside Mordor Macula displays an irregular, jumbled landscape (Fig. 9). The limb displays large relief in this polar area. Stereo topography reveals a number of irregular depressions (Fig. 3), and one near the 270° longitude position is 10 km below the average Charon elevation. We can also see pole-facing ridges which appear to have dark crests and dark material either exposed on their slopes or mass-wasted down from the dark unit at the crest.

Dark spots with well-defined edges can be seen on the floor of that deepest depression, which aren’t seen elsewhere on Charon. If there was a more substantial atmosphere on Charon (Stern et al., 2016), one might suppose they were aeolian collections of dark material, but that seems an unlikely explanation for these features. It is also unlikely that the shading is due to local topography casting shadows, as the incidence angle here is approximately 28°, which is a relatively high Sun angle.

Mordor Macula itself appears to have a very different color signature than the rest of Charon (Grundy et al., 2016b), but its structural history is difficult to discern. The stereo data show a very distinct topographic ridge that appears to bound the majority of the dark red material of Mordor Macula running from approximately longitude 215° (where the topographic data begin in the west) to longitude 21° where the ridge (and the dark red material) is interrupted by the Dorothy Gale crater. If this arcuate ridge is the rim of a crater, then one would expect this crater rim to continue on the east side of Dorothy Gale, but no topographic ridge is seen.

Either the topographic ridge is the rim of a large ancient impact, which has large sections of its rim erased by subsequent impacts or activity, or the suspiciously arcuate ridge has nothing to do with impact cratering, and is simply another ridge created by the tectonic forces that shaped Oz Terra.

### 2.4. Non-encounter hemisphere features

**Fig. 10:**
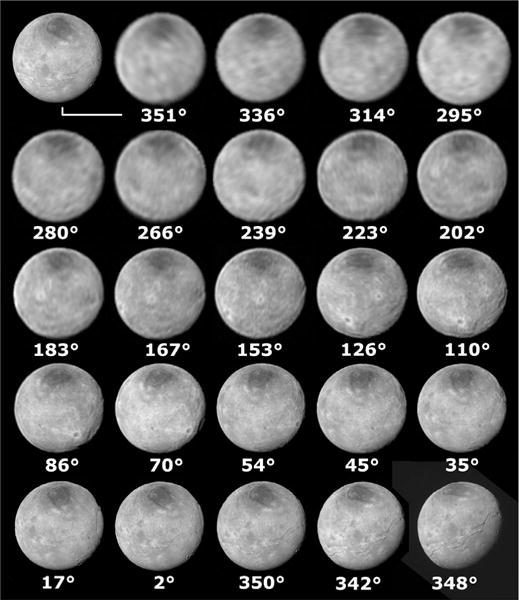
LORRI coverage for one rotation before New Horizon’s closest approach. Numbers below each image show the approximate center east longitude on Charon for each image. The upper left image without a label is the image at 350° reprojected to match the image at 351° in order to help match features. These images are interlaced deconvolved versions of the images from the following LORRI sequences: PC_MULTI_MAP_A_18_L1AH (351°), PC_MULTI_MAP_B_1 (336°), PC_MULTI_MAP_B_2 (314°), PC_MULTI_MAP_B_3 (295°), PC_MULTI_MAP_B_4 (280°), NAV_C4_L1_CRIT_33_02 (266°), PC_MULTI_MAP_B_6 (239°), NAV_C4_L1_CRIT_34_02 (223°), PC_MULTI_MAP_B_8 (202°), PC_MULTI_ MAP_B_9 (183°), PC_MULTI_MAP_B_10 (167°), NAV_C4_L1_CRIT_35_03 (153°), PC_MULTI_MAP_B_12_L1AH_02 (126°), NAV_C4_L1_CRIT_36_02 (110°), NAV_C4_L1_ CRIT_37_02 (86°), PC_MULTI_MAP_B_15_02 (70°), PC_MULTI_MAP_B_16_02 (54°), PC_MULTI_MAP_B_17_02 (45°), PC_MULTI_MAP_B_18_02 (35°), PCNH_MULTI_ LONG_1D1_02 (17°) PC_MULTI_LONG_1d2a_02 (2°), and C_LORRI_FULLFRAME_1 (350°). The two bottom right images are both from the time near closest approach. The image on the left is a deconvolved mosaic of LORRI images from the C_LORRI sequence and the image on the right is MVIC image MC0_0299176432.

**Fig. 11:**
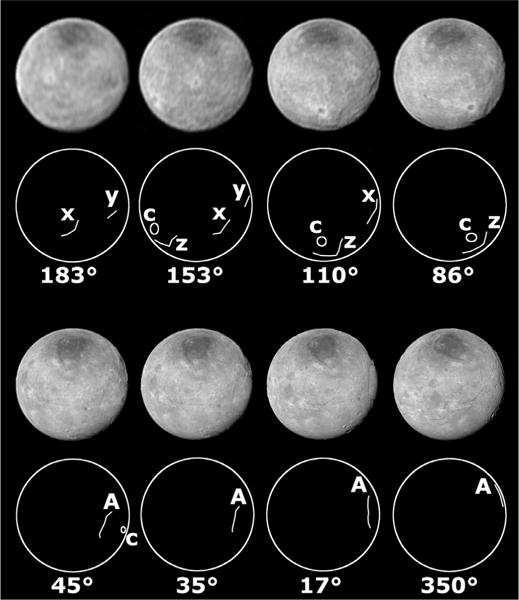
These images are the same as in Fig. 10. The diagrams show the locations of assumed features. Argo Chasma is labeled ‘A.’ The large bright-rimmed crater near Argo is labeled ‘c.’ There are three large unnamed lineations, ‘x,’ ‘y,’ and ‘z’ on the non-encounter hemisphere that can be observed in several images.

Approach images from LORRI show a variety of potential tectonic features in the non-encounter hemisphere (Figs. 10 and 11 primarily the 223° through 86° images). Argo Chasma can be seen in the 45° center latitude image, but when it is centered on the disk in the 86° center latitude image, it can’t be seen, which is probably due to the lower resolution. To the east of Argo is a large (~100 km) bright-rayed crater (circle marked ‘c’ in Fig. 11), and to the east and south of that crater in the 86° and 110° center latitude images are some dark linear features (at 110 and 125°E, lines in Fig. 11) more than 150 km long that are likely to be large scarps or chasmata given the lighting geometry. Similarly, the 110° center longitude image shows a dark linear feature on the eastern limb at 174°E, 13°N that is probably another large scarp or depression that is more than 200 km long (line marked ‘x’ in Fig. 11).

### 2.5. Tectonic strikes

**Fig. 12:**
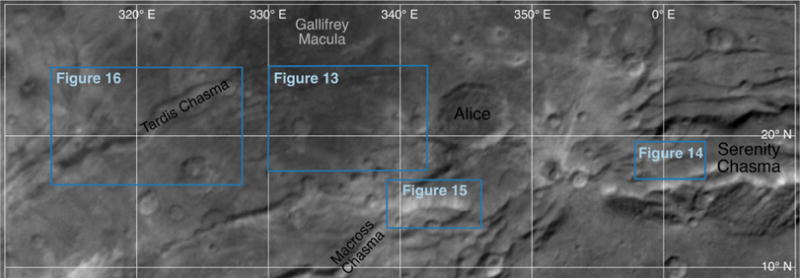
Example tectonic features in the inset of Figs. 13–16. This image is a mosaic of the LORRI images from the C_LEISA_LORRI_1 sequence.

**Fig. 13:**
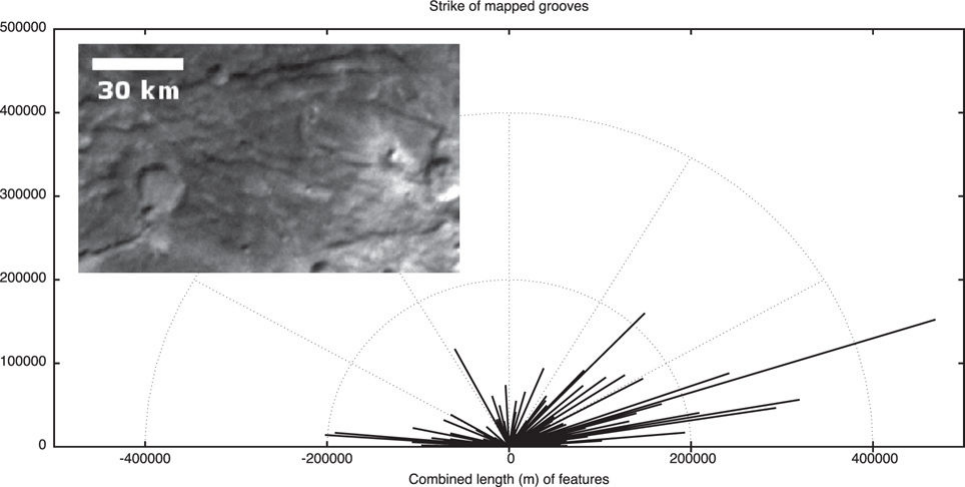
The strike of groove segments mapped on Fig. 4. The inset shows grooves west of Alice crater; Sun from the north.

**Fig. 14:**
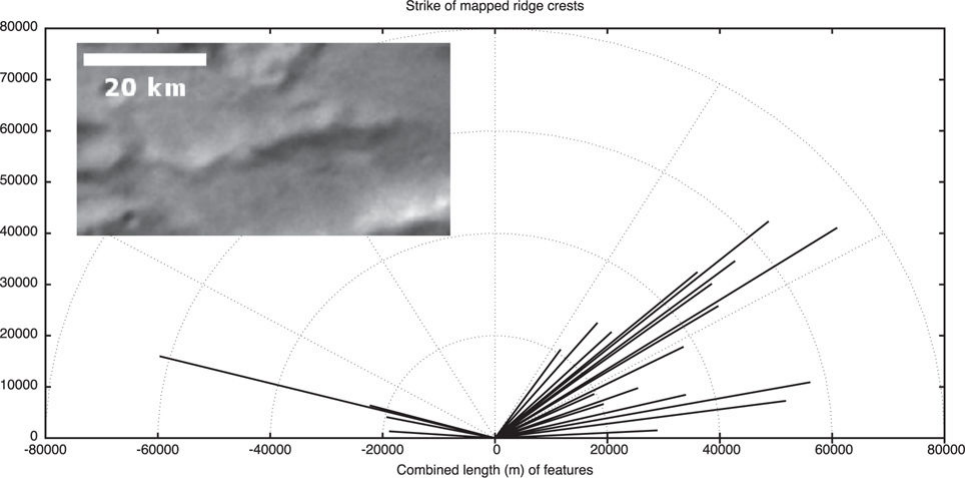
The strike of ridge crest segments mapped on Fig. 4. The inset shows a typical ridge in Serenity Chasma; Sun from the north.

**Fig. 15:**
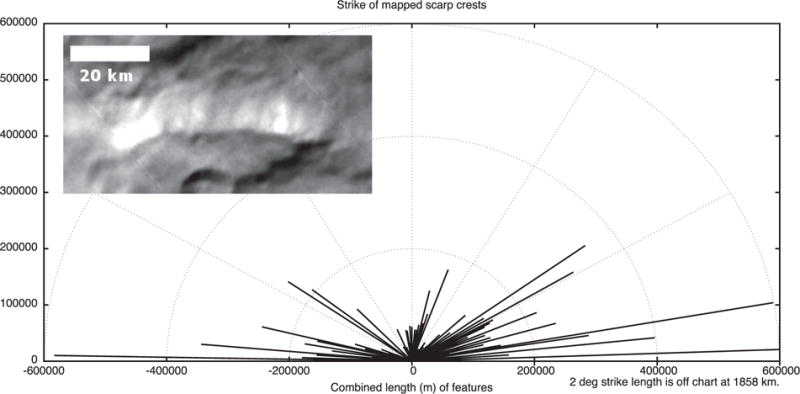
The strike of scarp crest segments mapped on Fig. 4. The inset shows a north-facing scarp just south of Alice crater; Sun from the north.

**Fig. 16:**
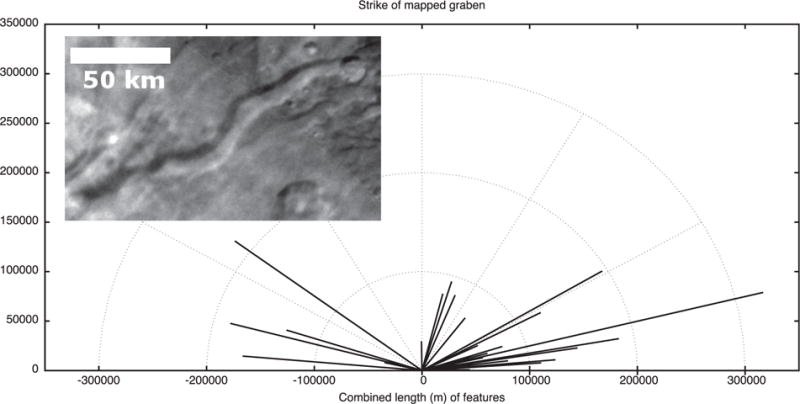
The strike of graben segments mapped on Fig. 4. The inset shows Tardis Chasma, a typical graben on Charon; Sun from the north.

The features mapped in Figs. 4 and 5 can be compiled and their strikes analyzed. The four major groups of features we measured in Oz Terra are grooves (Fig. 13), ridge crests (Fig. 14), scarp crests (Fig. 15), and graben (Fig. 16), examples of these features are seen in (Fig. 12). While this analysis displays a variety of strike directions, there is a distinct majority of strikes that trend approximately east-west indicating that the majority of extension was in the north-south direction in Oz Terra.

## 3. Results

### 3.1. Timing of faulting

Numerous craters are observed to superpose the low-latitude scarps and chasmata, ranging in size from a resolution limited lower bound of ~1–2 km in diameter, up to several craters ~55 km in diameter, which occur at low latitudes and do not appear modified by later tectonics. This abundance of craters (including some larger ones) is consistent with an old age of ~4 Ga found for Charon’s northern and southern terrains (Moore et al., 2016a; Singer et al., 2016). This age estimate is based on crater distributions and assumed impact rates (Greenstreet et al., 2015 Greenstreet et al., 2016).

Vulcan Planum appears to embay Oz Terra from the south, and has at least one possible outlier within Oz Terra (Spencer et al., 2016; Moore et al., 2016a). This outlier starts at a cratered area near the boundary of Oz Terra and Vulcan Planum, and extends northwards to about 40° N (Fig. 8). This area is elevated, but does not have steep flanks like the mid-latitude blocks in this area. This particular area has a few grooves that cross it, but does not have any large graben like the areas to the east and west of it in the tectonic map (Fig. 4). We interpret this as a flow that occurred during the emplacement of Vulcan Planum which found a low spot and flowed northwards. We conclude that Vulcan Planum is slightly younger than the terrains of Oz Terra. Given that at least the main resurfacing of Vulcan Planum is an ancient event (due to the high density of craters), this is again consistent with Oz Terra’s deformation early in Charon’s history. A few craters may be cut or modified by later faulting, but their formation may also have been affected by the topography of the target, or later mass wasting (Singer et al., 2016).

### 3.2. Elastic lithosphere

**Fig. 17:**
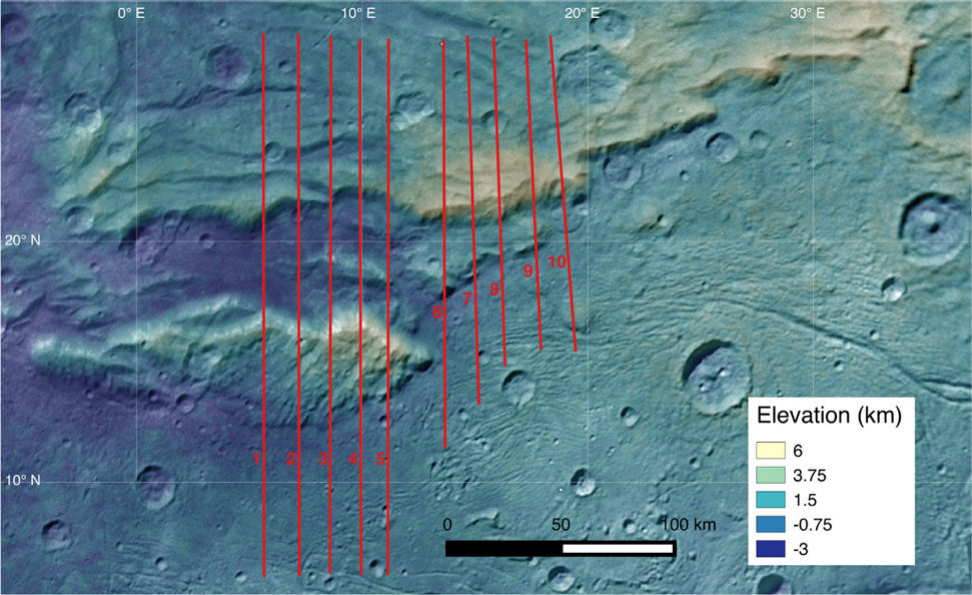
A colorized terrain model of Serenity Chasma can be seen with numbered profiles marked, starting with profile 1 on the left.

We measured topographic profiles across the classic rift valley shape of Serenity Chasma (Fig. 17) and used them to place bounds on the elastic thickness of Charon’s lithosphere.

**Fig. 18:**
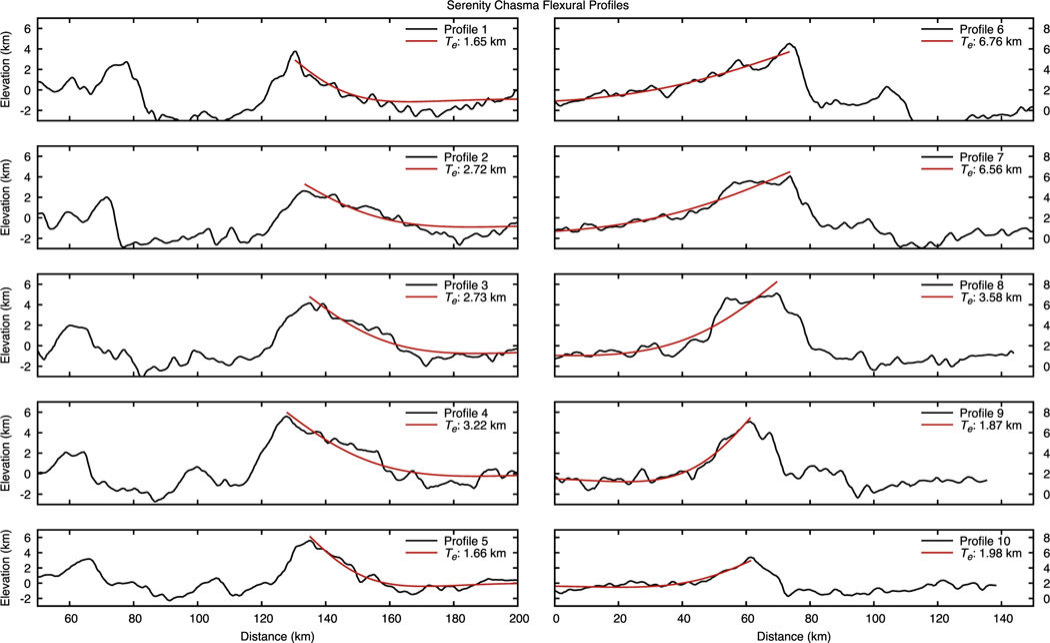
Profiles from Fig. 17 with flexural fits superimposed. Each profile is labeled with the best-fit elastic thickness *T_e_*. Flexural fitting procedure and parameter values adopted are the same as in (Peterson et al., 2015) except for the surface gravity.

The largest vertical offset on Serenity Chasma is more than 6 km (e.g. Fig. 18, profile 6). Apparent dip angles are 30° or less. This is low for a normal fault, whose typical slopes are 60°, and most likely implies mass wasting of the original scarp. In some places the footwall appears back-tilted (e.g. the south side of profiles 1–5) at an angle of a few degrees. This could be due to flexural uplift, but might also be simply bookshelf or other faulting (Jackson and White, 1989; Pappalardo et al., 1997) and rotation in response to extension.

To investigate this issue further, we carried out flexural fits to a sub-set of rift-flank profiles (Fig. 18) using the same approach that Peterson et al. (2015) used for Ariel, and the same parameter values except for the gravity, 0.29 m s^−2^. The mean elastic thickness *T_e_* we infer for Charon is 2.5 km. This value is similar to that inferred at Arden Corona, Miranda (Pappalardo et al., 1997). It is less than the *T_e_* values of 5–7 km for Ithaca Chasma on Tethys (Giese et al., 2007) and 3.8–4.4 km from a rift zone on Ariel (Peterson et al., 2015) (although some of our fits are in that range). The Charon value and those of the others are all larger than the 0.9 km for a rift flank on Ganymede (Nimmo et al., 2002). These differences may reflect real and interesting differences in the formation of extensional tectonics across the outer solar system.

**Fig. 19:**
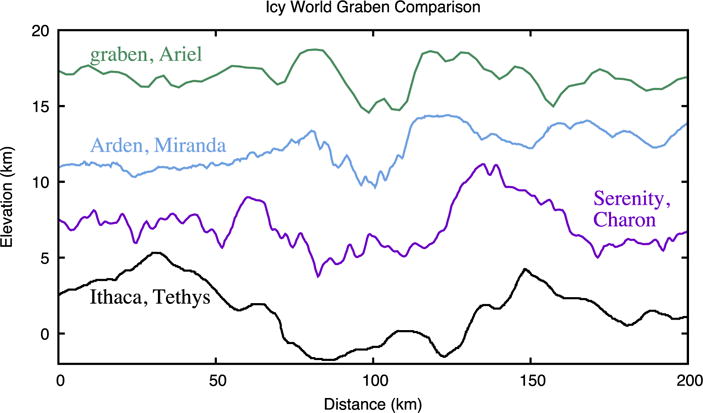
A comparison of profile 3 (Fig. 17) from Serenity Chasma compared to similar profiles from Ithaca Chasma, Tethys (Giese et al., 2007); Arden Corona, Miranda (Pappalardo et al., 1997); and graben on Ariel (Peterson et al., 2015).

The elastic thickness recorded would be the lowest since the time of deformation, and is thus reflective of thermal conditions around the time of Serenity Chasma formation, and not the present day. While Serenity Chasma is about as deep as Ithaca Chasma, it is only half as wide (Fig. 19), while Serenity is about twice as wide as the rifts at Miranda’s Arden corona (Pappalardo et al., 1997) and graben on Ariel (Peterson et al., 2015).

Assuming an initial dip angle of 30–60° (typical of normal faults) and a throw of 4 km (estimated from the terrain models), the total horizontal extension across the graben is 5–14 km. Taking the average graben width to be 60 km, the local stretching factor is then 1.08–1.25. A back-tilting angle of 3° implies a stretching factor of 1.05 for an initial 45° dip angle (Jackson and White, 1989), roughly consistent with this estimate.

The stresses induced by the observed topography are given by

(1) $$\sigma_{\text{max}}\approx \frac{3\rho gh}{8\pi^{2}}{\left(\frac{\lambda }{T_{e}}\right)}^{2}$$

where *λ* is the graben width, *h* the maximum throw, *ρ* the density, *g* the gravity, and here we assume that *T_e_ < λ*/*π* (Jackson and White, 1989). The bounding faults must be strong enough to withstand these stresses, because otherwise the faults would move in such a way as to reduce the topography (Jackson and White, 1989). Taking *λ* = 50 km, *T_e_* = 2.5 km and *h* = 4 km, we obtain stresses of about 16 MPa. That is roughly the frictional stress on a fault at 100 km depth on Charon, from *ρghμ* based on assuming a coefficient of friction, *μ* of 0.6 (Beeman et al., 1988), a density of 0.92 g/cc, and gravity of 0.288 m/s^2^. We are therefore faced with an inconsistency: the brittle thickness is more than an order of magnitude larger than the elastic thickness, whereas the two values are expected to be comparable. The most likely resolution is that the elastic thickness exceeds 2.5 km, and that the rift flank profiles are not recording flexure, they may just be undergoing tilting/rotation in response to the extension they underwent (e.g. bookshelf faulting).

Charon’s craters are another witness to the thickness of the lithosphere. If the elastic thickness was only a few kilometers, then craters larger than 20 km in diameter should exhibit up-bowed floors. This behavior is not seen in the topography of craters of that size and larger on Charon (Figs. 2 and 3), another indication that the lithosphere of Charon has been relatively thick for a good portion of Charon’s observable geologic history.

**Fig. 20:**
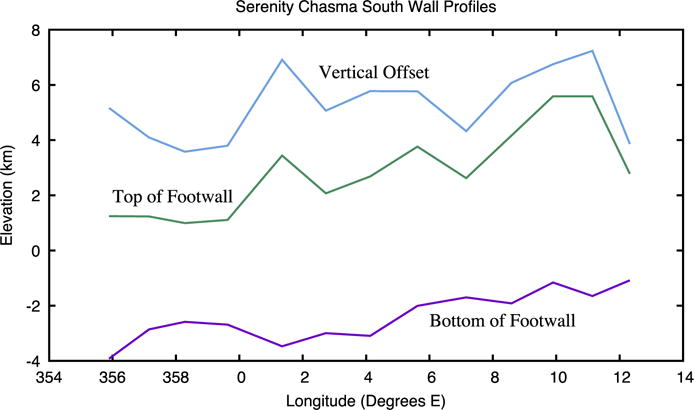
These traces show the local maximum (top of footwall) and minimum (bottom of footwall) topography along the southern wall of Serenity Chasma (profiles shown in Fig. 17). The difference between these two levels is also plotted as the vertical offset across the scarp.

The vertical offset along individual fault segments behaves in a similar manner to normal faults elsewhere. Fig. 20 shows the displacement profile for the southern bounding scarp at the western end of Serenity Chasma. The total fault segment length is about 250 km and the maximum displacement-to-length ratio is about 0.02. The latter value is essentially identical to normal faults on Europa (Nimmo and Schenk, 2006) and resembles terrestrial displacement:length ratios (Dawers and Anders, 1995). It does not support the suggestion that displacement:length ratios should scale with gravity (Schultz et al., 2006). The fault segment length is large compared to many terrestrial normal faults. This suggests that Charon has a brittle layer thickness comparable to or larger than the ~15 km thickness characterizing typical terrestrial continental crust (Jackson and White, 1989).

Our general assumption is that the scale of faulting is controlled by the thickness of the brittle layer—a good example of this is mid-ocean ridges on Earth, where fault dimensions and spacing depend on the spreading rate (Carbotte and Macdonald, 1994). Therefore, in a qualitative sense, these large faults and wide rifts on Charon are suggestive of a thick brittle layer.

### 3.3. Global extension

As described above, the rift geometry of Serenity and Mandjet Chasmata allow estimates of the extension across them. The minimum extension assumes the bounding normal faults dip steeply (~60°), in which case the depth of the rift is a close approximation to the extension across it. If Serenity Chasma is assumed to be 300 km long with an minimum depth of 3 km (Fig. 20) and Mandjet Chasma is taken to be 450 km long with a depth of 5 km, together they represent an areal increase of ~3000 km^2^, or an areal strain of ~0.3% over the northern half of Charon’s encounter hemisphere.

In addition to Serenity and Mandjet, we evaluated the 23 other largest scarps in Oz Terra, which pushes this minimum areal strain estimate to ~1%. These scarps all had relatively similar plan-form widths of 3–5 km. Eleven of them were longer than 100 km, and they contributed about 80% of the area to the total extension calculation. If we examine the four longest (347 km, 245 km, 300 km, 450 km), they contributed 46% of the area, indicating that the majority of the extension in Charon’s Oz Terra comes from the few large faults.

This estimate of a 1% areal strain can be conceptually decomposed into a 0.5% linear strain in two orthogonal directions. Such a minimum linear strain, *ε* ~ 0.5%, corresponds to elastic extensional stresses of ~*εE* = 50 MPa for a Young’s modulus *E* for water ice of 10 GPa. This is sufficient to cause motion on pre-existing faults to depths of several hundred kilometers. The widths of Serenity and Mandjet Chasmata themselves are consistent with (but do not require) normal faults that penetrate to tens of kilometers. Deep normal faulting is in general an enabling condition for volcanic eruptions, as long as there is sufficient buoyancy or magma pressure to drive the eruption. Furthermore, on an icy body with a subsurface ocean, extensional surface stresses occur simultaneously with pressurization of the ocean (Manga and Wang, 2007). It is therefore not unreasonable to link the early, strongly extensional tectonic state of Charon’s northern terrain and rift belt with the apparently cryovolcanic resurfacing of Vulcan Planum to the south (Moore et al., 2016a; Spencer et al., 2016). We conclude that similarly strong extensional tectonics likely affected the southern portion of Charon’s encounter hemisphere, before the emplacement of Vulcan Planum materials.

Global extensional stress states generally imply global volume changes (Collins et al., 2010). In Charon’s case both temperature changes and phase changes (melting/freezing, hydration/dehydration) can play a role. The volume thermal expansion coefficient for water ice varies from zero at ~75 K to 1.6 × 10^−4^ K^−1^ at 250 K (Petrenko and Whitworth, 1999), so it is difficult for even a large temperature change to result in a radial expansion of 0.5%, or equivalently, a volumetric expansion of ≃ 1.5%. Moreover, Charon is likely about one third rock by volume based on its mean density (McKinnon et al., 2016; 2017), and silicates generally have much lower thermal expansion coefficients than ices (by more than an order of magnitude), although potential temperature changes within an inner core due to radiogenic heating can exceed 10^3^ K (Desch, 2015; Malamud and Prialnik, 2015). We conclude that temperature changes alone are not responsible for the global expansion evidenced by Charon’s tectonics, but they could be an important contributor.

Dehydration/hydration reactions may have been important for Charon’s evolution. Dehydration in particular, as represented by the serpentine breakdown reaction:

(2) $$\underset{\text{serpentine}}{{\left(\text{Mg},\text{Fe}\right)}_{3}{\text{Si}}_{2}O_{5}{\left(\text{OH}\right)}_{4}}\to \underset{\text{olivine}}{{\left(\text{Mg},\text{Fe}\right)}_{2}{\text{SiO}}_{4}}+\underset{\text{opx}}{\left(\text{Mg},\text{Fe}\right){\text{SiO}}_{3}}+2H_{2}O$$

which results in a local volume increase of ~3% when H_2_O is released as water, and ~6% overall if the water migrates to a level where it freezes.^2^ The maximum global volume increase is then limited by Charon’s rock volume fraction to 1–2%. Charon’s rock in this case would of necessity be sequestered in a core, in order to realize dehydration temperatures of ~500 °C. Long-term radiogenic heating, the only plausible mechanism to drive such dehydration, is expected to reach peak temperatures over longer time intervals, greater than one billion years after core formation (Malamud and Prialnik, 2015; Desch, 2015), so it is unclear if dehydration of a previously hydrated rock core is a suitable explanation for Charon’s rather early tectonics. Long-term cooling (perhaps in the latter half of Solar System history) might also be expected to result in at least partial rehydration (retrograde metamorphism), and thus surface compression, which is not seen.

Because of Charon’s small size, whether or not radioactive decay in the interior is sufficient to cause melting and ocean formation depends on poorly known parameters such as the amount of NH_3_ present, the degree of porosity and the propensity of the ice to overturn (e.g. Desch et al., 2009; Malamud and Prialnik, 2015). The putative Charon-forming impact is unlikely to have imparted enough energy to Charon to produce a global subsurface ocean (Canup, 2005; 2011). Subsequent tidal heating is a potential additional source of energy to drive ocean formation (Barr and Collins, 2015). Any such ocean is unlikely to have survived to the present day (Hussmann et al., 2006). From the tectonic point of view, what matters most is the extent to which any ancient ocean re-froze.

Early freezing of an internal ocean could easily have driven Charon’s global expansion (Stern et al., 2015; Moore et al., 2016a). As is well known, freezing of water results in ~8.5% volume expansion. For an ice shell overlying an ocean, it can be shown that the areal expansion in response to progressive freezing of the ice shell is given by this geometric relation

(3) $$2\frac{\Delta d}{R}\frac{\Delta \rho }{\rho_{i}}$$

where *R* is the radius of the body (606 km), Δ*d* is the increase in shell thickness, *ρ_i_* is the ice density, Δ*ρ* the density contrast between ice and water, and Δ*ρ*/*ρ_i_* ≈ 0.085. Thus, an areal strain of 1% can be accomplished by an increase in shell thickness of about 35 km. Hence, even a relatively thin, ancient ocean which then refroze could have generated strains in the inferred range. The extent and freezing time of such an early ocean requires further study, but the studies cited above suggest that such an ocean is a plausible outcome of Charon’s evolution.

Both relative and absolute age data would be helpful in distinguishing between different hypotheses for the origin of the extensional tectonics. For instance, any successful hypothesis has to explain the apparently older nature of the tectonics relative to the emplacement of Vulcan Planum. The absolute age of the tectonic features is currently uncertain by at least a factor of 2 (Greenstreet et al., 2015 Greenstreet et al., 2016), with a likely age in the range from ~4 to ~2 Ga. Future work to reduce this uncertainty would be important; for instance, ocean re-freezing is unlikely to have been complete within the first ~0.5 Ga of solar system history.

### 3.4. Origins of tectonic orientation

It was argued in Section 2.5 that there appear to be preferred orientations of tectonic features on Charon. Such an apparent preference may in part be due to an observation bias because of the lack of complete imaging coverage. Nonetheless, ocean freezing by itself is expected to generate isotropic stresses, and thus not produce a preferred orientation. Tidal and/or despinning stresses are one possibility for oriented tectonic patterns (Barr and Collins, 2015; Rhoden et al., 2015), but the predicted patterns should be globally symmetrical about the equator or prime meridian, which is not consistent with the observed distribution. Lateral heterogeneities in ice shell properties are another possibility. A third possibility is that Charon’s ice shell experienced true polar wander (Nimmo and Matsuyama, 2007), which would reorient existing lineations while potentially also generating new tectonic features. Whether any of these hypotheses is consistent with the available observations will be the subject of future work.

### 3.5. Comparison to Pluto

Pluto and Charon may be a binary system, but their structural geology and surface morphologies are very different. Charon’s water-ice outer shell is rigid at surface temperatures, resulting in the brittle deformation features described here. The diverse ice species on Pluto (CO, N, CH_4_, etc.) have less strength, and so the softened morphologies of Pluto’s Sputnik Planitia (White et al., 2016) and its nitrogen glaciers (Howard et al., 2016) simply aren’t possible on Charon’s surface. Furthermore, the presence of an atmosphere and the potential seasonal cycling of volatiles on Pluto leads to degradation and erosion of features (Moore et al., 2016b) that is not seen on Charon.

## 4. Conclusions

The global characteristics of Charon’s tectonics are striking: (1) extension dominates over the entirety of Charon’s northern terrain up to and including the scarps that border Vulcan Planum, whose resurfaced units bury the flanks of the rifts; (2) there is no evidence for compressional faulting or strike-slip faulting; (3) the extension is inferred to be relatively ancient (up to ~4 Ga), based on the superposition of many craters and Vulcan Planum units, which are themselves relatively heavily cratered; and (4) the roughly polygonal extension across the northern terrain does not indicate a preferred direction of extensional stress, whereas the roughly east-west alignment of the major chasmata implies major north-south extension across the entire structural belt represented by the chasmata.

The widespread patterns of extensional tectonics detailed here suggest that Charon experienced a few kilometers of radial expansion. One possible mechanism for driving such expansion is the refreezing of a subsurface ocean. Charon’s silicate interior likely contained enough energy to initially melt part of an overlying ice shell, especially given the evidence for ammonia in Charon’s compositional makeup (Grundy et al., 2016a). As that heat was lost and the mostly-water ocean froze, the inevitable volume expansion from the phase change would have driven expansion of the crust, leading to extensional tectonics and perhaps cryovolcanism. This assumed sequence of events indicates that all of Charon’s tectonic activity would derive from this ocean-freezing period. Once freezing was complete, Charon became a much less dynamic world, and has been tectonically ‘quiet’ for most of the subsequent history of the solar system.

## Footnotes

1 All names in this work are informal, and follow the informal names in Moore et al. (2016a).

2 Based on the molar volumes of water at STP (18.018 cm^3^/mol), ice (19.65 cm^3^/mol), and the magnesian end members antigorite, forsterite, and enstatite (111.63, 43.66, and 31.35 cm^3^/mol, respectively), adjusted for the modest core pressures likely for Charon (50–300 MPa) and an antigorite breakdown temperature of ~600 C.
